# Unlocking the healing power of psilocybin: an overview of the role of psilocybin therapy in major depressive disorder, obsessive-compulsive disorder and substance use disorder

**DOI:** 10.3389/fpsyt.2024.1406888

**Published:** 2024-06-11

**Authors:** Sandra Szafoni, Piotr Gręblowski, Klaudia Grabowska, Gniewko Więckiewicz

**Affiliations:** ^1^ Students’ Scientific Circle in Department of Psychiatry, Faculty of Medical Sciences in Zabrze, Medical University of Silesia, Katowice, Poland; ^2^ Department of Psychiatry, Faculty of Medical Sciences in Zabrze, Medical University of Silesia, Katowice, Poland

**Keywords:** psilocybin, psilocybin-assisted psychotherapy, major depressive disorder, obsessive-compulsive disorder, substance use disorder, nicotine addiction, alcohol use disorder, psychedelics

## Abstract

Resistance to traditional treatment methods is still a major obstacle in modern psychiatry. As a result, several studies are currently being conducted to find effective alternatives to traditional therapies. One of these alternatives is psilocybin, a psychedelic substance that has been tested in clinical trials as an adjunct to psychotherapy. These studies focus on patients with major depressive disorder (MDD), obsessive-compulsive disorder (OCD) and substance use disorder (SUD), particularly alcohol and nicotine dependence. This article looks at the current understanding of psilocybin, including data from clinical trials conducted, psilocybin’s mechanism of action, its safety and the level of risk associated with it.

## 1 Introduction

The emergence of modern psychiatry has led to the development of a multitude of psychotherapeutic and pharmacological strategies. Nevertheless, despite these advances, a certain percentage of patients still struggle with mental health problems due to treatment resistance. This percentage is relatively high, ranging from 20–60% according to various estimates, which poses a major challenge for mental health professionals (1). Consequently, researchers are actively exploring effective alternative approaches. Their aim is to find out whether any substances can expand the available treatment options. It is crucial to underscore that these novel medical interventions are intended to serve as an alternative treatment for patients for whom conventional therapies have not brought enough improvement. They are not intended to replace first-line therapy, but rather to provide an additional option for individuals who have not achieved success with established approaches.

Interestingly, some of the substances under investigations are classified as schedule I drugs (2), what is meant to point to high potential for addiction and lack of approval for medical use. One of them are psychedelics, in which case the prohibition came in the 1960s and 1970s with the rise of the counterculture movement and the growing popularity of psychedelics. This was due to concerns about their safety, potential for abuse and risks to public health (3). However, even before their delegalization, there were a growing number of reports pointing to their therapeutic potential, which can be attributed to their unique properties (3). In recent years, the interest and need for scientific research in this area has increased. As recent scientometric analysis indicates, we can even speak of a “psychedelic renaissance” marked by a significant increase in scientific publications and clinical trials exploring the therapeutic potentials of them (4). As a result, some countries have introduced legislative reforms to facilitate clinical trials and the therapeutic use of psychedelics under controlled conditions. This happened with psilocybin, which was later recognized by the Food and Drug Administration as a “Breakthrough Therapy”. This status was first awarded for Treatment-Resistant Depression and later for Major Depressive Disorder (MDD) (5). This designation demonstrates that research on psilocybin therapy has shown promising results in the treatment of certain conditions and underscores the Food and Drug Administration’s commitment to accelerate the development and approval of this treatment. Psilocybin, also known as the ‘magic mushroom’ due to its hallucinogenic properties, is found in nearly 200 species of mushrooms worldwide (6). Although psilocybin was only isolated in 1957 by the chemist Albert Hofmann, it has a long history spanning centuries, if not millennia (6, 7). Evidence from artifacts, cave paintings and cultural traditions strongly suggests that psilocybin-containing mushrooms have been known and used by various civilizations and indigenous cultures throughout human history (6, 7). Today, despite prohibition, psilocybin is still used illicitly by individuals for similar purposes. Some users take psilocybin to achieve altered states of consciousness and sensory experiences during musical events, while another group of users are primarily looking to experience ego dissolution and gain a new perspective. Through such experiences, it is hoped to gain new insights, a heightened sense of connection and a re-evaluation of one’s perspective on life, consciousness and existence. The latter effects are also used in a medical context and offer the possibility of profound changes in the perception of oneself and the surrounding world.

In view of the growing interest and the increasing number of research studies available in this field, we would like to provide a comprehensive summary of the current state of knowledge about psilocybin-assisted psychotherapy. The paper was synthesized based on English-language peer-reviewed publications retrieved from databases such as PubMed, EMBASE, Cochrane, and Web of Science. Articles published not earlier than in 1996 were included. Due to the narrative nature of the review, tools characteristic of other types of reviews, such as Preferred Reporting Items for Systematic Reviews and Meta-Analyses (PRISMA), were not employed. Our article will focus on the implications for the treatment of depression, obsessive-compulsive disorder and substance abuse, particularly alcohol abuse and nicotine addiction. First, we will briefly and clearly outline the potential mechanisms of action of psilocybin and how it relates to the pathophysiological basis of each disorder. We will then present the research results, information and conclusions. We will discuss the safety and potential risks of psilocybin, taking into account its addictive potential. Finally, we will determine whether psilocybin could be a viable alternative to traditional psychiatric interventions. By analyzing existing research and considering its therapeutic potential, we will provide an answer to this question.

## 2 Psilocybin-assisted psychotherapy’s mechanism of action

**Figure 1:**
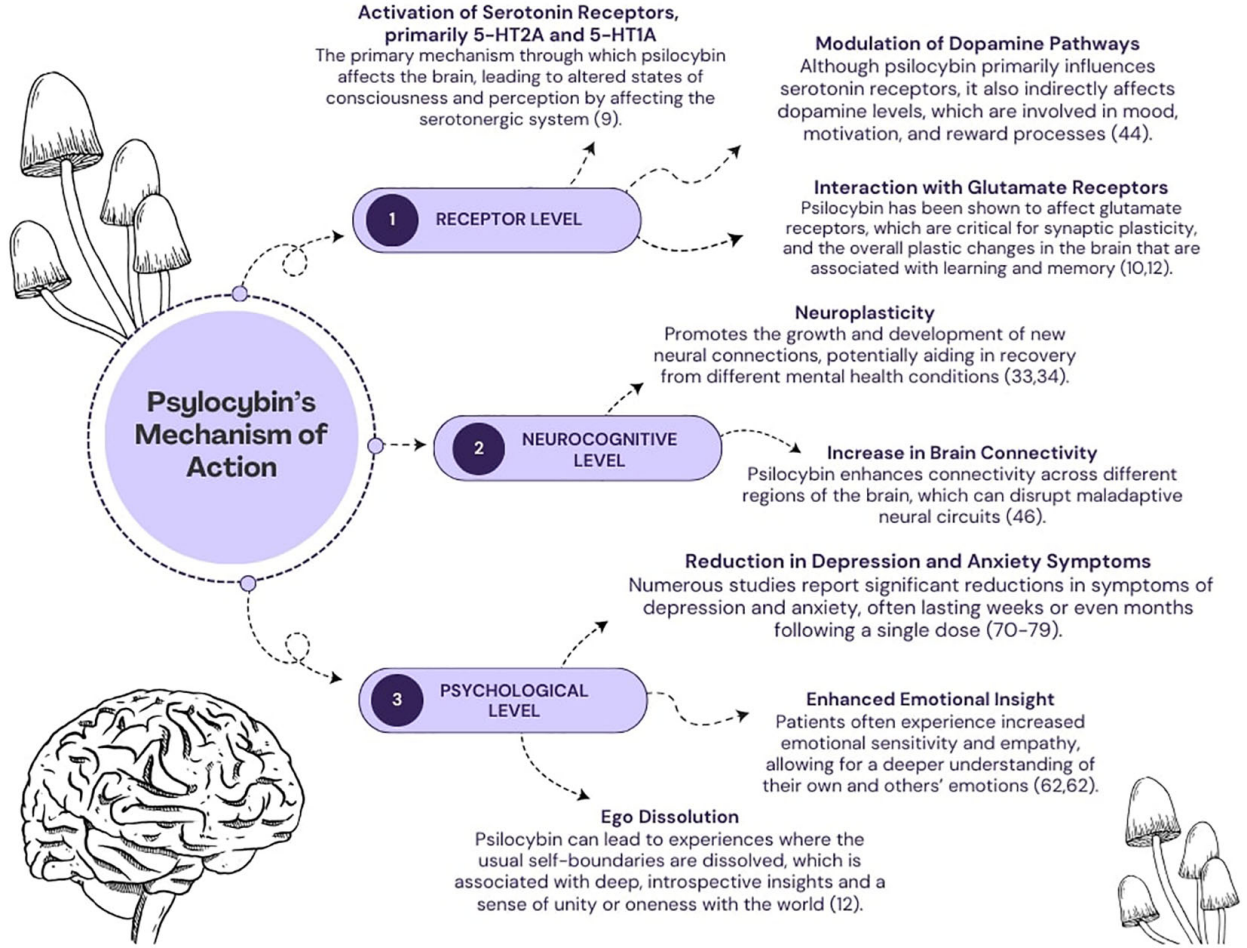
A diagram depicting several mechanisms of psilocybin’s action. The effects of its action are categorized into three levels: (1) pharmacological, (2) neurocognitive, (3) psychological.

The exact mechanism underlying the effects of psychedelics, including psilocybin, remains complicated and has not yet been fully elucidated. To date, researchers have proposed a variety of models and theories to explain the unique properties of psychoactive substances. It is worth noting that these concepts are not mutually exclusive but, on the contrary, complementary to a certain extent. They provide different perspectives and insights into the underlying mechanisms and thus collectively contribute to our evolving understanding of their effects. In general, the effects of psilocybin can be considered on three primary levels (**Figure 1**): pharmacological, neural and psychological (8).

### 2.1 Pharmacological level

To date, the main viewpoint explaining the pharmacological effects of psilocybin has been its agonism at serotonergic receptors, particularly 5-HT2A and to a lesser extent 5-HT1A (9). However, the interaction between psilocybin and the glutamatergic system has come under increasing scrutiny, particularly in patients with alcohol problems (10). Glutamate is the most important excitatory neurotransmitter in the central nervous system, acting on a variety of receptors and playing a crucial role in synaptic transmission and the regulation of cognitive processes. However, excessive activation of glutamate receptors can lead to excitotoxicity, a phenomenon characterized by a pathological increase in intracellular calcium ions that can trigger an undesirable cascade of reactions. This cascade leads to neuronal damage, the release of various inflammatory mediators and finally to apoptosis. When considering the glutamatergic system in the context of psilocybin-assisted therapy, it is important to emphasize that this psychedelic substance does not affect it directly. Instead, it causes changes in the expression and function of one of the glutamate receptors – Metabotropic Glutamate Receptor 2 (mGlu2) - through its interaction with the 5-HT2A receptor, which is activated by psilocybin (11, 12). At this point, it should be noted that the activation of mGlu2 receptors reduces the excessive release of glutamate, which may be important from a pathophysiological point of view in various psychiatric disorders. Thus, the process of excessive glutamate release is associated with the reward system and addictions, including alcohol dependence (13). In general, alcohol acts as a depressant on the central nervous system by increasing the activity of inhibitory Gamma-Aminobutyric Acid (GABA) receptors while suppressing the activity of excitatory glutamate receptors. When alcohol consumption is stopped abruptly, the glutamatergic system becomes overly active, which can lead to neuronal hyperactivity and withdrawal symptoms. These symptoms may include anxiety, trembling of the limbs or even seizures in the patient (14). Therefore, activation of mGlu2 receptors after psilocybin ingestion may help these patients alleviate withdrawal symptoms, reduce cravings for alcohol and limit the occurrence of the harmful phenomenon of excitotoxicity. When alcohol is abused, excessive amounts of glutamate are often released in the brain, which can lead to over-activation of neurons and potentially damage or death — a process known as excitotoxicity. MGlu2 activation has a neuroprotective effect by limiting excessive glutamate secretion and reducing neuronal activity. In the context of alcoholism, regulation of this pathway may help to reduce alcohol cravings and withdrawal symptoms, which is beneficial in the treatment of addiction. It therefore appears that controlling this neurochemical signaling pathway may be critical to the effectiveness of addiction treatment. However, in disorders such as OCD or depression, studies suggest that disturbances in the function of the glutamatergic system may play an important role (15, 16). In OCD, high levels of glutamate in the brain have been shown to contribute to obsessive thoughts, compulsive behavior and anxiety, particularly in brain regions such as the anterior cingulate cortex (ACC) and the supplementary motor area (SMA) (17). In relation to depression, the role of the glutamatergic system and modulators of glutamate receptors has been discussed extensively in the literature as potential treatments. One such substance is esketamine, an antagonist of one of the glutamate receptors – N-Methyl-D-Aspartate (NMDA) (18). It has shown rapid and sustained antidepressant effects in patients with treatment-resistant depression and major depressive disorder, raising high therapeutic hopes for other modulators of the glutamatergic system, including psilocybin (19–21). With regard to the serotonergic system itself, which probably plays a central role in the effects observed after psilocybin ingestion, the structural and chemical similarity between its metabolite psilocin and serotonin should be emphasized. In other words, this implies the possibility of binding to serotonergic receptors, which are densely localized in numerous brain regions, including those closely associated with the manifestation of depression and anxiety symptoms (22). Interestingly, this aspect should be of interest not only in the context of research on MDD and OCD, but also with regard to the addictions currently under discussion. It is not uncommon for individuals with substance use disorders (SUD) to experience depression and anxiety-like symptoms, especially during abrupt withdrawal (23). In addition, all of the patient groups we have mentioned suffer from chronic stress. This has been documented in the literature and is associated with changes in hormone levels and dysregulated function of the hypothalamic-pituitary-adrenal (HPA) axis. The HPA axis is a neuroendocrine system responsible for regulating the stress response and maintaining internal balance in the face of changing environmental conditions (24–26). The potential effect of psilocybin on this axis would be via the activation of serotonergic receptors in the hypothalamus, which trigger the secretion of corticotropin-releasing factor (CRF) and thus cause the activation of the HPA axis. Interestingly, this is consistent with reports that psilocybin can transiently increase cortisol and ACTH levels even without a stress test, with levels returning to baseline after a few hours (27).

Given the important role of cortisol in the proper functioning of learning and memory processes, an increase in cortisol may be particularly relevant in a therapeutic context. First of all, it should be mentioned that all of the above-mentioned patient groups struggle with deficits in various memory components (28, 29). The temporary increase in cortisol levels can support the patient’s formation of new crucial beliefs by promoting learning and memory processes and change their attitude towards various past life experiences. In this context, it is crucial to emphasize that non-adaptive and inflexible beliefs interfere with therapy and affect emotions and behavior in patients with depression, obsessive-compulsive disorder and drug addiction (30–32). For example, an alcohol-dependent person may have a fixed behavioral pattern of automatically resorting to alcohol consumption when faced with a strong stressful stimulus in order to relieve emotional tension or distance themselves from the problem at hand. A similar problem can occur in people suffering from obsessive-compulsive disorder, in which certain situations trigger cognitive or behavioral automatisms. Similarly, people with depression often have a self-propelling cycle of negative thoughts that can lead to a lack of specific, required action for the individual. Breaking these patterns requires the formation of new neural pathways that gradually become dominant over time in a given situation. These changes, supported by mechanisms that promote neuroplasticity, are among the most important in the context of psilocybin-assisted psychotherapy (33). The phenomenon of neuroplasticity, which characterizes the brain’s ability to adapt and change in response to a range of experiences, reflecting the dynamic adaptability of neural circuits, is closely related to the psychoplastogens model. This model assumes that various substances known as psychoplastogens, e.g. classical psychedelics, influence the nervous system through a kind of autoregulatory feedback loop, thus promoting neuroplasticity (34). In short, these substances have been shown to increase synaptic growth and dendritic complexity and increase connections between neurons (35). This enhanced neuroplasticity is attributed to their postsynaptic effects in the medial prefrontal cortex, specifically in the fifth layer, where they stimulate glutamate release and activate α-amino-3-hydroxy-5-methyl-4-isoxazolepropionic acid (AMPA) receptors (11). As a result, this process triggers the Brain-Derived Neurotrophic Factor - Tropomyosin Receptor Kinase B (BDNF-TrkB) and mTOR signaling pathways, leading to upregulation of genes associated with neuroplasticity and synaptic protein synthesis (36).

The final pharmacological mechanism is the modulation of the inflammatory response, which is closely linked to the HPA axis. This issue is related to the anti-inflammatory model, according to which stimulation of a specific population of 5-HT2A receptors can induce changes at the epigenetic level (37). According to this concept, epigenetic changes inhibit the pro-inflammatory effects of interleukin-6 (IL-6) and tumor necrosis factor-alpha (TNF-α), thereby suppressing the inflammatory process in the body. This suggestion is extremely interesting given the role that inflammation plays in the etiology of various psychiatric disorders (38–40). However, the overall therapeutic effect cannot be attributed to the potential anti-inflammatory properties alone. Otherwise, one could speak of a high efficacy of the use of anti-inflammatory drugs in these disorders, which has not been proven so far.

### 2.2 Neurocognitive level

The therapeutic potential of psilocybin is also underpinned by its complex interaction with neurocognitive mechanisms. We can understand these mechanisms through the lens of recent neuroimaging studies and the corresponding theoretical models. In the field of psychedelic substances, some of the most commonly discussed or well-known frameworks that combine research findings from neuroimaging techniques include thalamo-cortical filter theory (also known as the cortico-striato-thalamo-cortical (CSTC) model), the relaxed beliefs under psychedelics (REBUS) model, and the claustro-cortical circuit (CCC) model (8). The CSTC model shows how psychedelics disrupt the sensory filters in the brain, leading to an overload of sensory information. According to this concept, the prefrontal cortex normally controls these filters, but psychedelics reduce its inhibitory control over the thalamus, leading to sensory overload (41). Several neural mechanisms are involved in this reduced thalamic gating, including agonism of 5-HT2A and antagonism of NMDA receptors. In addition, dopaminergic and GABAergic projections are involved, leading to an increase in activity (42). Furthermore, studies using PET and fMRI have shown altered brain activation and connectivity under psychedelics, which is consistent with the CSTC model (43, 44). However, the efficacy of this model is limited given the mixed results of behavioral tests in relation to reduced thalamic gating during psychedelic experiences (45, 46). In the field of psychedelic-assisted psychotherapy, the REBUS model offers a fairly integrated perspective (47). It states that psychedelics such as psilocybin, through their action on 5-HT2A receptors, increase the brain’s sensitivity to prediction errors, leading to flexible beliefs and increased sensitivity to sensory input (48, 49). At lower doses, these effects are manifested in perceptual phenomena such as “breathing walls” or “visual trails” especially with eyes closed (47, 49). In contrast, higher doses of psychedelics affect higher-level brain regions, including the default mode network (DMN), which is associated with self-related processing. The reduced activity of the DMN correlates with the dissolution of the ego and a loosening of fundamental beliefs. However, the different results on DMN activity may be due to methodological differences and the dynamic nature of the psychedelic experience (48, 50, 51). Interestingly, the REBUS model also incorporates the entropic brain hypothesis, which posits that psychedelics increase connectivity between typically unconnected brain networks, explaining synesthetic experiences and the unpredictability of the psychedelic journey (52, 53). From a therapeutic perspective, it suggests that psychedelics can temporarily loosen maladaptive beliefs that occur in all of the disorders we have considered. This allows the individual to gain new perspectives and insights. The deep sense of connection and self-transcendence during the psychedelic experience challenges established beliefs and promotes belief change.

The final model - the CCC model - examines the role of the claustrum, a subcortical brain region rich in 5-HT2A receptors and connected to different cortical areas (54, 55). This model hypothesizes that psychedelics activate the 5-HT2A neurons in the claustrum, leading to disruption of normal network states in the brain and a disconnection between the prefrontal regions and the claustrum, resulting in decreased cognitive control. Psilocybin has been shown to affect the claustrum and correlate with experiences such as ineffability and decreased coupling with other cortical networks, which may contribute to the decreased executive functions observed under psychedelic influence (56). However, the CCC model lacks specificity regarding which brain circuits are affected by psychedelic-induced changes in the claustrum, so advanced imaging techniques are needed to clarify this. Therefore, while the model offers insights, it may not fully explain all subjective effects of psychedelics.

### 2.3 Psychological level

In this novel therapeutic area, the psychological effects are as important as any other aspect. This is confirmed by the growing number of scientific sources that emphasize the wide range of positive psychological effects. Although this type of intervention is usually delivered as cognitive-behavioral therapy, the literature underscores that further research is needed to determine which approach is the most appropriate (57).

In general, the structure of psilocybin-assisted psychotherapy is divided into three main phases: the initial preparation, the session with the psychoactive substance and the integration of the experience after the session.

Such a method ensures increased safety and effectiveness of the procedure. A prepared patient who knows what to expect during the psychedelic session and how to manage their emotions can respond better to the unconventional, intense experience. This in turn helps to minimize the occurrence of potentially dangerous reactions from the participant. Sessions with a qualified, appropriately trained psychotherapist following a psilocybin session, which often reshape the participant’s perspective on life, allow for a deeper understanding of all that has happened during the session and a new way of looking at oneself, leading to altered responses to future events. In the field of psilocybin-assisted psychotherapy, the terms “set” and “setting” are inextricably linked to the therapeutic process (58). The “setting’ encompasses the inner attitude and expectations of the person undergoing therapy, including elements such as personal intention, emotional state and willingness to engage with the experience. Therefore, it is also directly related to the aforementioned adequate preparation for psychotherapy. The term “setting” refers to the external environment in which the therapy takes place. It should be designed to provide safety, serenity and comfort so that the individual feels supported on their journey. In addition, the use of music plays a central role in this environment, often using continuous ambient sounds that are conducive to introspection and emotional processing. The selection of therapeutic playlists aims to enrich the participant’s journey and enhance the introspective facets of the psilocybin experience. However, it should be noted that the selection of music remains an ongoing investigation that requires further research and refinement (59). Regarding the psychological effects observed in participants after a psychedelic session, it should be noted that some of them are difficult to measure and subjective. Nevertheless, people who have undergone psilocybin-assisted therapy often describe these experiences as deeply introspective, as they were able to explore their inner thoughts and feelings with great clarity (60, 61). This heightened introspection often leads to a deep psychological insight that helps sufferers to better understand themselves and the factors that contribute to their condition (60, 61). In addition, this substance can lead to changes in personal beliefs and values, allowing individuals to rethink their approach to life and their priorities (61). It can also increase motivation and give them a new drive to overcome personal challenges. In addition, the psychedelic experience increases self-efficacy and empowers individuals to take control of their lives and make positive changes. Intriguing psychological effects have been observed in individuals struggling with alcohol addiction who undergo psilocybin-assisted therapy. Scientific reports suggest that psilocybin improves the adaptability of self-related cognitive processes, reduces shame-related self-criticism, improves emotional regulation and reduces cravings for alcohol (62). In summary, the psychological dimensions of psilocybin-assisted therapy illustrate its profound effects on mental health. By facilitating an examination of self-perception and worldview, psilocybin catalyzes a transformative process that can significantly alleviate psychological problems.

## 3 Major depressive disorder

Depressive disorders are among the most common mental disorders in the world. The World Health Organization (WHO) estimates that it affects 5% of the world’s population (63) and is a major contributor to more than 700,000 suicides per year (64). Although effective treatments exist, the incidence of treatment-resistant depression, typically defined as a lack of therapeutic success after two attempts with antidepressants in monotherapy, is still widespread and is estimated to range from 30% to even 70%, as there is no standard definition (65–67). This naturally leads to the search for new antidepressant therapies, even if this means resorting to illegal substances that were considered medically useless in the past. Depression appears to be the primary focus of current scientific research on the medical applications of psilocybin and has shown the most success to date. Most of the research is randomized controlled trials (**Table 1**) that focus on one or two sessions of psilocybin administration in a psychologically supportive environment. The subjects of the interventions in the aforementioned studies mostly suffer from major depressive disorder (68–73, 77) and symptoms of depression and anxiety due to a life-threatening cancer (74–76). A detailed summary of the research on this topic is presented in the form of a table. Overall, psilocybin administration sessions with some form of psychological support appear to be very effective in treating symptoms of depression and have been reported to occur as early as one week after treatment (69, 70, 72) or even immediately after the acute effects have worn off (74), which is a very promising and desirable feature as most previous antidepressant treatments take some time to reach full efficacy. The therapeutic effect has been reported to last up to 12 months after treatment (71) The profile of adverse effects of psilocybin that has emerged in these studies appears to be mild, with headache, nausea and dizziness being most commonly reported and serious adverse effects being rare.

**Table 1:** Summary of the research regarding psilocybin use in the treatment of Major Depressive Disorder as well as anxiety and depressive symptoms in patients with life-threatening cancer.

Authors and year	Type of Study	Sample Size	Characteristic of participants	Intervention	Results	Conclusions
Goodwin et al. 2022 (68)	RCT	233	Patients with treatment-resistant depression	Single dose of 25 mg, 10 mg and 1 mg as a control with psychological support, measuring response with MADRS at 3 weeks and 12 weeks.	Significant reduction of depression score in patients receiving 25 mg dose compared to 1 mg over a 3 week period.	Psilocybin along with psychological support seems to be effective over a period of 3 weeks in decreasing depression scores in patients suffering from treatment-resistant depression but larger and longer trials, including comparison with existing treatments are needed.
Carhart-Harris et al. 2021 (69)	RCT	59	Patients with long-standing, moderate-to-severe MDD	Patients received two separate doses of 25 mg of psilocybin 3 weeks apart plus 6 weeks of daily placebo (psilocybin group) or two separate doses of 1 mg of psilocybin 3 weeks apart plus 6 weeks of daily oral escitalopram (escitalopram group); all the patients received psychological support. Response was measured with QIDS-SR-16	The mean changes in the scores from baseline to week 6 were -8.0 ± 1.0 points in the psilocybin group and -6.0 ± 1.0 in the escitalopram group, for a between-group difference of 2.0 points (95% confidence interval (CI), -5.0 to 0.9) (P = 0.17). A QIDS-SR-16 response occurred in 70% of the patients in the psilocybin group and in 48% of those in the escitalopram group, for a between-group difference of 22 percentage points (95% CI, -3 to 48); QIDS-SR-16 remission occurred in 57% and 28%, respectively, for a between-group difference of 28 percentage points (95% CI, 2 to 54).	This trial did not show significant difference in antidepressant effect between psilocybin and escitalopram. Larger and longer trials are required to compare psilocybin with established antidepressants.
Davis et al. 2021 (70)	RCT	24	Patients with MDD	Two psilocybin sessions (session 1: 20 mg/70 kg; session 2: 30 mg/70 kg) were given in the context of supportive psychotherapy (approximately 11 hours). Effects were evaluated in Hamilton Rating Scale for Depression (HAM-D) and in the Quick Inventory of Depressive Symptomatology-Self-Report (QIDS-SR)	17 participants (71%) at week 1 and 17 (71%) at week 4 had a clinically significant response to the intervention (≥50% reduction in GRID-HAMD score), and 14 participants (58%) at week 1 and 13 participants (54%) at week 4 were in remission (≤7 GRID-HAMD score). The QIDS-SR documented a rapid decrease in mean depression score from baseline to day 1 after the session, which remained statistically significantly reduced after 4 weeks.	Study suggests that psilocybin combined with therapy is effective in treating MDD, nevertheless more studies with bigger samples are required.
Gukasyan et al. 2022 (71)	Follow-up, RCT	24	Patients with MDD	Two psilocybin sessions (session 1: 20 mg/70 kg; session 2: 30 mg/70 kg) were given in the context of supportive psychotherapy (approximately 11 hours). Effects were evaluated in Hamilton Rating Scale for Depression (HAM-D) and in the Quick Inventory of Depressive Symptomatology-Self-Report (QIDS-SR). All patients attended the follow-up visits through a 12-month period.	Large decreases from baseline in GRID-HAMD scores were observed at 1-, 3-, 6-, and 12-month follow-up Treatment response (≧̸50% reduction in GRID-HAMD score from baseline) and remission were 75% and 58%, respectively, at 12 months. No serious adverse effects have been reported.	Study suggests that psilocybin-assisted therapy might be effective in treating depressive symptoms up to 12 months following acute intervention.
Carhart-Harris et al. 2016 (72)	Non-RCT (open-label trial)	12	Patients with treatment-resistant depression (MDD)	Two sessions of 10 mg and 25 mg psilocybin in a supportive setting. Change in depressive symptoms has been assessed mainly with QIDS and also with BDI, STAI-T, SHAPS, HAM-D, GAF	QIDS depression scores were significantly reduced from baseline to 1 week and 3 months post-treatment, with the maximum effect at 2 weeks. BDI and STAI scores were reduced at 1 week, 3 months after treatment. SHAPS scores were reduced at 1 week and 3 months after treatment. HAM-D and GAF scores were reduced 1 week	Study brings preliminary evidence about safety and efficacy of psilocybin in treating treatment-resistant depression and shows that further studies, especially RCTs, are needed and justified.
Carhart-Harris et al 2018 (73)	Follow-up, Non-RCT (open-label trial)	20	Patients with treatment-resistant depression (MDD)	Two sessions of 10 mg and 25 mg psilocybin i a supportive setting. Change in depressive symptoms has been assessed mainly with QIDS and also with BDI, STAI-T, SHAPS, HAM-D, GAF. 6 month follow up.	19 patients completed all measures. QIDS depression scores were significantly reduced from baseline at all assessment points from 1 week and 6 months post-treatment, with the maximum effect at 2 weeks. BDI and STAI scores were reduced at 1 week, 3 and 6 months after treatment. SHAPS scores were reduced at 1 week and 3 months after treatment. HAM-D and GAF scores were reduced 1 week after treatment. No serious adverse effects were reported.	This follow-up seems to suggest that effects of single dose intervention with psilocybin might have a beneficial effect on patients with treatment-resistant depression up to 6 months after the session. This seems to warrant further randomized controlled trials.
Ross et al. 2016 (74)	RCT	29	Patients with life-threatening cancer and related anxiety and depression	Single dose psilocybin 0,3 mg/kg vs niacin, both with psychotherapy. Assessment with HADS-A, HADS-D, HADS-T, BDI, STAI-T and STAI-S	Immediate, substantial and sustained up to 7 weeks after dosing clinical benefits reflected in all of the measures used.	Single dose psilocybin intervention shows promise as a safe measure to fight cancer related depression and anxiety.
Griffiths et al. 2016 (75)	RCT	51	Patients with life-threatening cancer and related anxiety and depression	Very low, placebo-like dose of 1 or 3 mg/70 kg vs. high dose of 22 or 30 mg/70 kg administered in two sessions in a 5 weeks interval. Outcomes measured with GRID-HAMD, HAM-A assessed with SIGH-A	Higher dose psilocybin group showed clinically significant response and symptoms remission for two primary outcomes of GRID-HAMD-17 and HAM-A scores that were sustained at 6-month follow-up	Psilocybin seems to improve depression and anxiety symptoms and overall quality of life in a sustained way in patients with life-threatening cancer. Further research seems justified and needed.
Grob et al. 2011 (76)	RCT	12	Patients suffering from depression, anxiety and advanced-stage cancer.	Psilocybin (0.2 mg/kg) vs. niacin (250 mg) in two sessions spaced several weeks apart. Effects measured with BDI, STAI-S, STAI-T. POMS	Improvement in BDI at 6 months after treatment. STAI score significant reduction at 1 and 3 month after treatment	Study provides data about feasibility and safety of administering psilocybin to patients with advanced-stage cancer. Study shows some improvement in the patient’s symptoms which supports the need for further bigger research in this topic.
Rotz et al. 2022 (77)	RCT	52	Patients with MDD	Single dose (0.215 mg/kg) with psychological support. Assessment with MADRS and BDI after 14 days.	After the intervention symptom severity decreased by absolute 13 points compared to baseline, and 13,2 points 14 days after the intervention.	This trial shows that a single, moderate dose is effective in rapidly decreasing depression symptoms up to 14 days after the intervention. Larger and longer studies are needed.

RCT, Randomized Controlled Trial; MDD, Major Depressive Disorder; MADRS, Montgomery–Åsberg Depression Rating Scale; QIDS, Quick Inventory of Depressive Symptomatology; BDI, Beck Depression Inventory; STAI, The State-Trait Anxiety Inventory; SHAPS, Snaith-Hamilton Pleasure Scale; GAF, Global Assessment of Functioning; SIGH-A, Structured Interview Guide for the Hamilton Anxiety; HAM-A, Hamilton Anxiety Rating Scale; POMS, Profile of Mood States.

## 4 Alcohol use disorder

Worldwide, around 237 million men and 46 million women suffer from alcohol use disorders (78). Alcohol abuse has a negative impact on the health of the individual and also has social consequences. Every year, 3 million people die from alcoholism, which accounts for 5.3% of all deaths (78). Therefore, it is crucial to reduce alcohol consumption in society and find an effective treatment method for addiction.

Current research on psilocybin therapies mainly focuses on the combination of behavioral therapy with two psilocybin sessions (78). Some studies introduce a comparison group receiving a placebo (79). The following studies (**Table 2**) show that patients participating in concurrent behavioral and psilocybin therapies are more effective in maintaining sobriety (78). Interestingly, patients showed a lower frequency of heavy drinking episodes (79). In addition, psilocybin as an adjunct to therapy sessions increases patients’ confidence in their ability to stay sober. The effects of therapy last up to 36 weeks (79). Further research is needed, but the results are promising.

**Table 2:** Summary of the research regarding psilocybin use in the treatment of Alcohol Use Disorder.

Authors and year	Type of study	Sample size	Characteristic of participants	Intervention	Results	Conclusions
Bogenschutz et al. 2015 (79)	RCT	10	Alcohol-dependent patients	4 weeks of treatment without psilocybin, then psilocybin administered orally in one or two sessions	Abstinence increased significantly during the 36-week follow-up. Patients also declared a higher self-efficacy of abstinence	The study shows that the use of psilocybin in the treatment of alcoholism can help patients stay sober longer and help them feel self-efficacious in abstinence
O’Donnell et al. 2022 (80)	RCT	96	Alcohol-dependent volunteers	Oral dose 25 mg/70 kg psilocybin or 50mg diphenhydramine (active placebo) in each of the two sessions (at weeks 4 and 8). The dose may have been increased in the second session. at the same time, the patients participated in psychotherapy for 12 weeks	The percentage of days of heavy drinking during the 32-week study period was 9.7% for the psilocybin group and 23.6% for the diphenhydramine group. The psilocybin group also had lower average daily alcohol consumption	The study shows that using psilocybin along with behavioral therapy can reduce the number of days of heavy drinking and the overall amount of alcohol consumed

## 5 Nicotine addiction

Nicotine addiction is currently one of the main factors causing serious health problems such as respiratory cancers or Chronic Obstructive Pulmonary Disease (81). Treating the health effects of smoking is a significant burden on health care budgets (82). That is why it is so important to search for an effective treatment. In 2020 statistics showed that about 22.3% of the world’s population consumes nicotine. Most consumers are male: 36,7% of male population uses tobacco (81). In highly developed countries, nicotine use is declining, especially among the highly educated population. In contrast, the number of smokers is increasing in low- and middle-developed countries (83). In one study (**Table 3**), nicotine-dependent patients were given two or three doses of psilocybin. The patients took part in cognitive behavioral therapy at the same time. The researchers tested the maintenance of non-smoking after 12 and 16 months. More than half of the subjects were successful, showing that psilocybin can facilitate smoking cessation. It may also prove useful in reducing the risk of relapse (84).

**Table 3:** Summary of the research regarding psilocybin use in the treatment of nicotine addiction.

Authors and year	Type of study	Sample size	Characteristic of participants	Intervention	Results	Conclusions
Johnson et al. 2017 (84)	open-label pilot study	15	Participants were 15 smokers - 5 women and 10 men with no history of serious mental illness. Their average age was 51 years. They smoked an average of 19 cigarettes a day for an average of 31 years. On average, they had 6 prior attempts to quit smoking	Patients took two to three sessions of psilocybin (first 20mg/70kg, second 30mg/70kg) in combination with cognitive behavioral therapy (CBT) for smoking cessation	10 participants remained abstinent from smoking after 12 months of follow-up. In the long-term 16-month follow-up, abstinence was confirmed in 9 patients	The results of the study suggest that psilocybin may help maintain long-term abstinence from nicotine

## 6 Obsessive-compulsive disorder

Obsessive-compulsive disorder (OCD) is a prevalent condition characterized by intrusive thoughts that provoke intense emotional tension. To alleviate/relieve this distress, individuals often engage in repetitive behaviors or rituals (85). It is estimated that around 2.3% of the population will develop OCD during their lifetime (86). Typically, the onset of the disorder occurs during adolescence or early adulthood and affects both genders equally, although among younger individuals, males are more frequently affected. Research exploring the potential use of psilocybin in treating OCD is still in its early stages; however, initial findings indicate promising prospects for further investigation. In a recent study (**Table 4**), the patients participated in four sessions involving varied doses of psilocybin. Remarkably, the participants reported a reduction in symptoms following the first or second session, with effects lasting longer than 24 hours. Moreover, the perceived therapeutic benefits seem to outweigh the relatively low risk of side effects associated with psilocybin administration (87).

**Table 4:** Summary of the research regarding psilocybin use in the treatment of OCD.

Authors and year	Type of study	Sample size	Characteristic of participants	Intervention	Results	Conclusions
Moreno et al. 2006 (87)	RCT	9	patients diagnosed with OCD, no other psychiatric disorders	4 sessions with low (100 mcg/kg), medium (200 mcg/kg) high dose (300 mcg/kg) psilocybin. Additionally after the first session, a randomized and double-blind very low dose was administered. A break between sessions lasted a minimum of one week. Sessions lasted 8 hours and were held in the hospital	the study received decreases in OCD symptoms in YBOCS scale (23–100%) after one or more sessions	Taking psilocybin under controlled conditions can reduce OCD symptoms in patients

RCT, Randomized Controlled Trial; OCD, Obsessive-Compulsive Disorder; YBOCS, Yale-Brown Obsessive Compulsive Scale.

## 7 Discussion

### 7.1 Interpretation of results

The study’s outcomes reinforced the hypothesis that psilocybin in medical context could prove extremely useful for certain patients suffering from various mental disorders. Currently, one of its most promising applications appears to be the treatment of major depression disorder, although its effectiveness in the treatment of alcohol use disorder and OCD also appears promising. These results are entirely to be expected given the extensive scientific literature highlighting psilocybin’s diverse and wide-ranging properties. The substance primarily acts on the nervous system through its strong affinity for serotonin receptors such as 5-HT2A and 5-HT1A, but recent research has also focused on its links to glutamate and its receptors. The main changes that occur under the influence of this psychoactive substance include changes at the epigenetic level, the promotion of neuroplasticity and changes in the activity of certain neuronal networks. It is important to emphasize that the effects observed in clinical studies are due to interactions at both neurobiological and psychological levels, without exposing patients to excessive health risks. The safety of psilocybin therapy is underlined by a study comparing its efficacy and side effects with those of escitalopram, the standard medication for depression. According to the results, patients with depression who took psilocybin experienced fewer side effects (69). Another significant advantage of psilocybin therapy is that the patient does not have to take the substance daily over a long period of time. This in turn could significantly reduce human error and premature discontinuation of therapy.

### 7.2 Limitations of the study

This article has several limitations, including methodological constraints. Relying on available English-language publications from specific databases, we may have overlooked some of the available studies. Furthermore, the results of the presented studies may have been overly generalized or extrapolated by us, given the small patient groups in some of the cited studies. Another issue undoubtedly lies in the problem of adequate result objectification and measurement limitations - many effects of psilocybin therapy are subjective and difficult to measure using standard psychometric tools, which may hinder the interpretation and assessment of therapy effectiveness. Equally important to note is the lack of long-term safety and side effects data of this type of therapy in all contexts, which should be taken into account in further research.

### 7.3 Psilocybin – current usage patterns and addiction concerns

In the realm of psilocybin’s classification as a Schedule I substance under prohibition, concerns remain about its addictive and harmful potential. However, in the case of this psychedelic, the available data suggest a low addictive potential due to two factors (88). Firstly, users quickly develop a tolerance to the substance due to rapid receptor desensitization (89, 90). Secondly, psilocybin acts primarily as an agonist of serotonin receptors, which differs from highly addictive drugs such as amphetamines or cocaine, which act directly on the mesolimbic dopaminergic pathway. When discussing the issue of addictive potential, it is relevant to consider what we know about today’s psilocybin users. Nowadays, the substance is used at events associated with rave and psychedelic trance (psytrance) culture due to its ability to induce altered sensory experiences (including synesthesia, heightened music perception, and visual hallucinations) (91, 92). At such events, appropriate music is played, accompanied by visual effects. While rave culture focuses on entertainment, psytrance culture focuses on spirituality and personal development (91, 92). Likewise, individuals engaged in various rituals involving “sacred plants” often aim to address a variety of psychological issues by ingesting psilocybin, N,N-Dimethyltryptamine (DMT), or ayahuasca. The “guides” or “supervisors” of the participants during such events are often people without appropriate training, and qualified medical professionals are not on-site, leading to numerous problems and risks for those seeking help. Therefore, the future adoption of standardized medical protocols and procedures that include the use of psilocybin-assisted psychotherapy in patients to treat specific conditions may prove to be a legal and safer alternative for individuals seeking such therapeutic interventions.

### 7.4 Implications for future research

Despite the existence of the previously mentioned findings, many issues still require further research and verification. Future studies should focus on longitudinal tracking of psilocybin treatment outcomes to assess the long-term efficacy and its safety. Understanding the duration and stability of the therapeutic effects can guide clinical application and patient follow-up strategies. Additionally, there is a need to delve deeper into the mechanisms through which psilocybin exerts its effects. Research focusing on the neurobiological changes, receptor interactions, and molecular pathways activated by psilocybin will provide insight not only into its therapeutic potential and possible side effects but may also contribute to our better understanding of the pathophysiology of mental illness in general. Moreover, the existing literature suggests the need for further exploration of its therapeutic possibilities. Beyond the conditions outlined previously, psilocybin-assisted therapy may hold broader implications. These diseases potentially encompass, for instance, social anxiety, generalized anxiety disorder, anorexia nervosa, post-traumatic stress disorder, or even negative symptoms of schizophrenia and personality disorders (93–97). Hence, it appears necessary to conduct additional studies in these areas and determine the effectiveness of psilocybin. Another aspect to scrutinize remains the therapy’s alterations. Contemporary scientific literature already contains preliminary suggestions for modifications that could prove beneficial. These include the inclusion of additional ingredients or the use of medical psilocybin in slightly different contexts. For instance, a recent study investigated one such proposal, in which patients received a small dose of methylenedioxymethamphetamine (MDMA) alongside psilocybin psychotherapy (98). The results indicated that patients had fewer challenging experiences while reporting increased feelings of self-compassion, love, and gratitude. At this point, it is worth considering the potential synergy between psilocybin and other rapid-acting antidepressants, including ketamine (99). Given the research outcomes regarding esketamine’s efficacy in depression, or the encouraging preliminary findings from studies involving arketamine, these matters unquestionably warrant deeper scientific exploration (100). Another proposal - using virtual reality (VR) techniques in therapy sessions, may have potential benefits, but its effectiveness has not been examined. However, researchers believe that such incorporation might help patients relax, relieve their anxiety, and enrich their overall experience (101). A recently presented, related approach explores the medical potential of psilocybin in combination with mindfulness training. The study hypothesized that such a combination may alter self-awareness and connectivity of the brain’s default network, translating into long-lasting results (102). Undoubtedly, further research is needed to investigate the therapeutic effects of psilocybin. This could help us to better understand psilocybin’s mechanisms of action, confirm these preliminary findings and determine the best methods for its integration into clinical practice.

### 7.5 Practical applications and recommendations

Based on our findings, we would like to outline several key issues and suggest specific actions that could contribute to better utilization of such therapies in the future. Firstly, to ensure optimal help for patients, therapists overseeing the process should undergo a standardized training program. This program should emphasize managing potential adverse effects and providing adequate support for participants before, during, and after psychedelic sessions. Additionally, it is crucial to define the prevailing therapeutic approach during these activities. The literature presents numerous proposals for therapeutic approaches, including well-established Cognitive-Behavioral therapy approaches such as Acceptance and Commitment Therapy or the related Accept, Connect, Embody model, as well as less scientifically grounded approaches like mindfulness-based therapies (103–108). Each approach brings unique perspectives, suggesting an integrative model might be the most appropriate. Flexible utilization of therapeutic techniques drawn from various approaches allows for tailoring the therapeutic tool to individual needs. However, additional research comparing the effectiveness of different approaches would be the most adequate solution and could provide us with the most information. Interestingly, an analysis of therapy outcomes indicates variations in the therapeutic doses of psilocybin across different conditions. For instance, in the treatment of drug-resistant depression, significantly higher doses of psilocybin (40 mg/70 kg) demonstrate greater efficacy compared to lower doses. Conversely, the maximum effective dose for secondary depression is lower (8.92 mg/70 kg) than for primary depression (24.68 mg/70 kg) (109). However, in the context of OCD therapy, higher doses of psilocybin have not shown increased effectiveness (87). Notably, in addiction studies referenced in this article, large doses of psilocybin have proven effective – specifically, 25 mg/70 kg in alcohol addiction treatment (80) and 20–30 mg/70 kg for nicotine addiction treatment (84). These findings underscore the importance of tailoring psilocybin dosages based on specific therapeutic objectives and patient profiles, highlighting the need for personalized treatment approaches in clinical practice. Furthermore, another challenge we face is the proper education of society regarding new psychiatric medications, including psilocybin. It is crucial to outline both the benefits and risks associated with psychedelic-assisted therapy. Appropriate informational campaigns could provide the necessary information to revise beliefs about this psychedelic. However, media reports and online information can instill in some individuals struggling with mental disorders a desire to seek out such interventions from unverified, illegal sources. Hence, conducting direct conversations in medical settings about this matter can be paramount. One objective will be to enhance awareness of the potential risks associated with self-administration of this substance. Another one will be to avoid the use of the services of self-proclaimed therapists or “spiritual guides” by patients. Appropriate set and setting as well as professional medical supervision are also necessary due to the fact there are potentially dangerous side effects that, although rare, may appear after administering psilocybin. In literature, we can find reports of elevated blood pressure, psychotic episodes, paranoia, extreme fear, and mood alterations, including euphoric mood (108, 110). It’s important to highlight that such effects may precipitate treatment-emergent affective switches in individuals with bipolar disorder (111). Hence, patients should be discouraged from seeking a substitute for psilocybin-assisted psychotherapy on their own to reduce the likelihood of their occurrence as much as possible.

### 7.6 Conclusions and their significance

**Figure 2:**
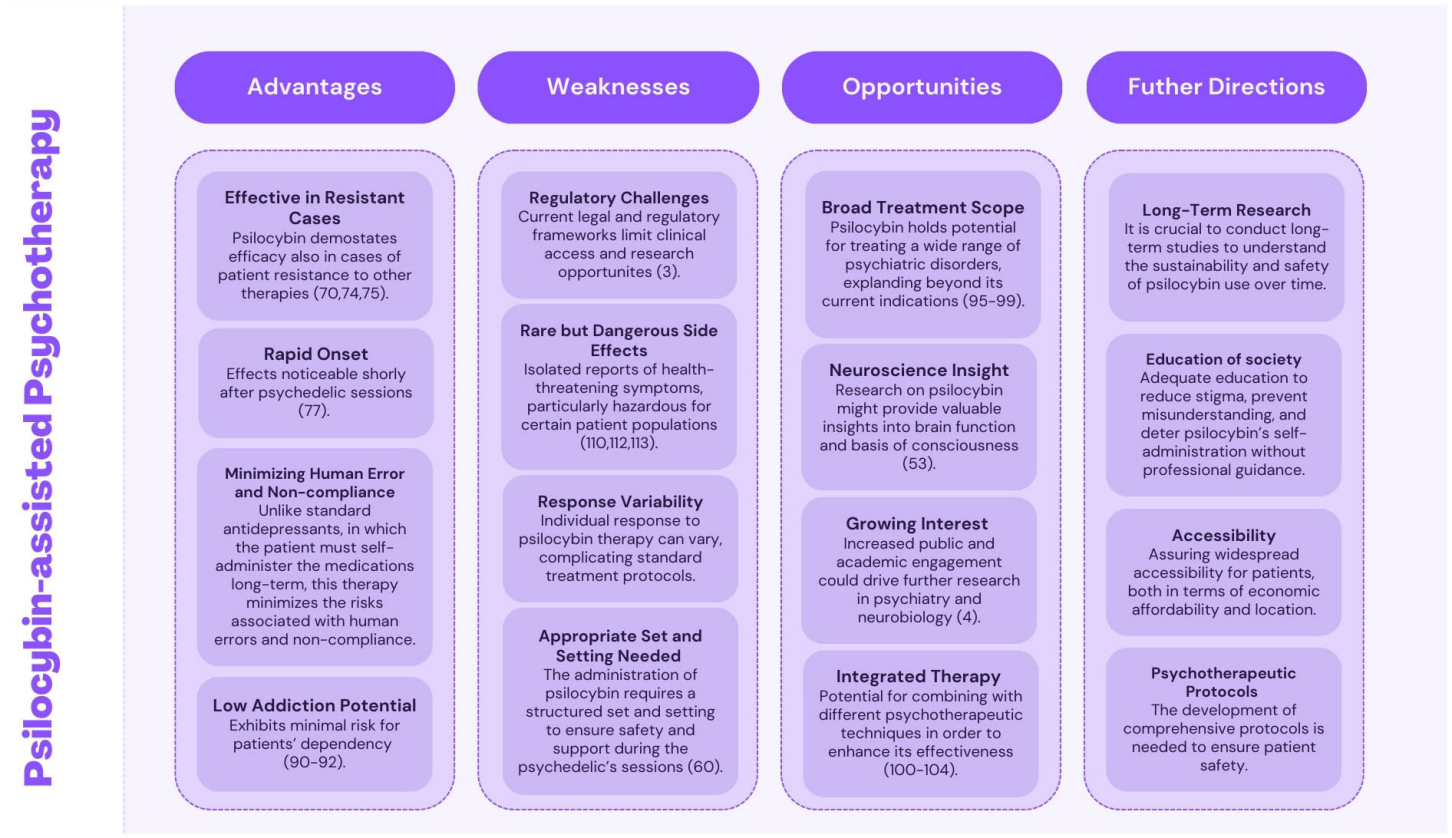
A schematic presenting the main outcomes derived from this literature review. To facilitate understanding, the conclusions have been organized into four categories: advantages, weaknesses, opportunities, and future directions.

The investigation into Psilocybin-Assisted Psychotherapy within this article showcases its potential as a significant therapeutic tool for addressing Major Depressive Disorder, Obsessive-Compulsive Disorder, and Substance Use Disorders, precisely targeting alcohol and nicotine dependencies. Nevertheless, as highlighted previously, the literature indicates the necessity of evaluating the effectiveness of this therapy in other psychiatric conditions. In general, the medical application of psilocybin appears to offer promising prospects for several reasons. Firstly, it rapidly alleviates symptoms of the conditions and maintains the durability of these effects over an extended period. Then, it represents a hybrid approach that marries conventional psychotherapy with psychedelic sessions involving controlled substance administration. As a result, this reduces the likelihood of human errors and patient discontinuation, often observed with standard antidepressant medication therapies. It is worth mentioning that, on the whole, patients have exhibited good treatment tolerance. However, some articles have reported isolated side effects cases that carried potential health risks. Lastly, but equally principal is providing hope to patients for whom traditional forms of therapy have fallen short. In addition, some study participants have already met these hopes. Its effectiveness in previously treatment-resistant cases likely stems from the multifaceted approach of this psychotherapy. As noted earlier, the utilization of this substance involves pharmacological, neurocognitive, and psychological levels. Thus, it provides a dual approach - it addresses the patient’s symptoms and supports substantial mental and emotional transformation. However, despite these positive indications, there remain areas requiring further investigation. Future research should focus on optimizing dosing protocols, understanding the full spectrum of psychological impacts, and expanding the scope of disorders treatable with psilocybin. Additionally, regulatory frameworks need to evolve to consider the therapeutic benefits of psychedelics like psilocybin while ensuring safe and controlled usage. In conclusion, psilocybin-assisted psychotherapy represents a novel and potentially transformative approach to psychiatric treatment, meriting significant attention and resource allocation for continued study and eventual integration into clinical practice (**Figure 2**).

## Author contributions

SS: Writing – review & editing, Writing – original draft, Project administration, Conceptualization. PG: Writing – review & editing, Writing – original draft. KG: Writing – original draft. GW: Writing – review & editing, Supervision.

## References

[1] Howes OD Thase ME Pillinger T. Treatment resistance in psychiatry: state of the art and new directions. Mol Psychiatry. (2022) 27:58–72. doi: 10.1038/s41380-021-01200-3 34257409 PMC8960394

[2] Sousa TR Rema J MaChado S Novais F. Psychedelics and hallucinogens in psychiatry: finding new pharmacological targets. Curr Top Med Chem. (2022) 22:1250–60. doi: 10.2174/1568026621666211201145800 34852736

[3] Nutt D. Psychedelic drugs—a new era in psychiatry? Dialogues Clin Neurosci. (2019) 21:139–47. doi: 10.31887/DCNS.2019.21.2/dnutt PMC6787540 31636488

[4] Solmi M Chen C Daure C Buot A Ljuslin M Verroust V. A century of research on psychedelics: A scientometric analysis on trends and knowledge maps of hallucinogens, entactogens, entheogens and dissociative drugs. Eur Neuropsychopharmacol. (2022) 64:44–60. doi: 10.1016/j.euroneuro.2022.09.004 36191546

[5] Heal DJ Smith SL Belouin SJ Henningfield JE. Psychedelics: threshold of a therapeutic revolution. Neuropharmacology. (2023) 236:109610. doi: 10.1016/j.neuropharm.2023.109610 37247807

[6] Nichols DE Walter H. The history of psychedelics in psychiatry. Pharmacopsychiatry. (2021) 54:151–66. doi: 10.1055/a-1310-3990 33285579

[7] Carod-Artal FJ. Hallucinogenic drugs in pre-Columbian Mesoamerican cultures. Neurología. (2015) 30:42–9. doi: 10.1016/j.nrl.2011.07.003 21893367

[8] van Elk M Yaden DB. Pharmacological, neural, and psychological mechanisms underlying psychedelics: A critical review. Neurosci Biobehav Rev. (2022) 140:104793. doi: 10.1016/j.neubiorev.2022.104793 35878791

[9] Madsen MK Fisher PM Burmester D Dyssegaard A Stenbæk DS Kristiansen S. Psychedelic effects of psilocybin correlate with serotonin 2A receptor occupancy and plasma psilocin levels. Neuropsychopharmacology. (2019) 44:1328–34. doi: 10.1038/s41386-019-0324-9 PMC6785028 30685771

[10] Domanegg K Sommer WH Meinhardt MW. Psychedelic targeting of metabotropic glutamate receptor 2 and its implications for the treatment of alcoholism. Cells. (2023) 12:963. doi: 10.3390/cells12060963 36980303 PMC10047550

[11] Vollenweider FX Kometer M. The neurobiology of psychedelic drugs: implications for the treatment of mood disorders. Nat Rev Neurosci. (2010) 11:642–51. doi: 10.1038/nrn2884 20717121

[12] Mason NL Kuypers KPC Müller F Reckweg J Tse DHY Toennes SW. Me, myself, bye: regional alterations in glutamate and the experience of ego dissolution with psilocybin. Neuropsychopharmacology. (2020) 45:2003–11. doi: 10.1038/s41386-020-0718-8 PMC7547711 32446245

[13] Alasmari F Goodwani S McCullumsmith RE Sari Y. Role of glutamatergic system and mesocorticolimbic circuits in alcohol dependence. Prog Neurobiol. (2018) 171:32–49. doi: 10.1016/j.pneurobio.2018.10.001 30316901 PMC6261463

[14] Dharavath RN Pina-Leblanc C Tang VM Sloan ME Nikolova YS Pangarov P. GABAergic signaling in alcohol use disorder and withdrawal: pathological involvement and therapeutic potential. Front Neural Circuits. (2023) 17:1218737. doi: 10.3389/fncir.2023.1218737 37929054 PMC10623140

[15] Vlček P Polák J Brunovský M Horáček J. Role of glutamatergic system in obsessive-compulsive disorder with possible therapeutic implications. Pharmacopsychiatry. (2018) 51:229–42. doi: 10.1055/s-0043-118665 28950396

[16] Wu K Hanna GL Rosenberg DR Arnold PD. The role of glutamate signaling in the pathogenesis and treatment of obsessive–compulsive disorder. Pharmacol Biochem Behav. (2012) 100:726–35. doi: 10.1016/j.pbb.2011.10.007 PMC3437220 22024159

[17] Biria M Banca P Healy MP Keser E Sawiak SJ Rodgers CT. Cortical glutamate and GABA are related to compulsive behaviour in individuals with obsessive compulsive disorder and healthy controls. Nat Commun. (2023) 14:3324. doi: 10.1038/s41467-023-38695-z 37369695 PMC10300066

[18] Khoodoruth MAS Estudillo-Guerra MA Pacheco-Barrios K Nyundo A Chapa-Koloffon G Ouanes S. Glutamatergic system in depression and its role in neuromodulatory techniques optimization. Front Psychiatry. (2022) 13. doi: 10.3389/fpsyt.2022.886918 PMC9047946 35492692

[19] Fava M Freeman MP Flynn M Judge H Hoeppner BB Cusin C. Double-blind, placebo-controlled, dose-ranging trial of intravenous ketamine as adjunctive therapy in treatment-resistant depression (TRD). Mol Psychiatry. (2020) 25:1592–603. doi: 10.1038/s41380-018-0256-5 PMC6447473 30283029

[20] Psiuk D Nowak EM Dycha N Łopuszańska U Kurzepa J Samardakiewicz M. Esketamine and psilocybin—The comparison of two mind-altering agents in depression treatment: systematic review. Int J Mol Sci. (2022) 23:11450. doi: 10.3390/ijms231911450 36232748 PMC9570062

[21] Wojtas A Bysiek A Wawrzczak-Bargiela A Szych Z Majcher-Maślanka I Herian M. Effect of psilocybin and ketamine on brain neurotransmitters, glutamate receptors, DNA and rat behavior. Int J Mol Sci. (2022) 23:6713. doi: 10.3390/ijms23126713 35743159 PMC9224489

[22] Ressler KJ Nemeroff CB. Role of serotonergic and noradrenergic systems in the pathophysiology of depression and anxiety disorders. Depress Anxiety. (2000) 12 Suppl 1:2–19. doi: 10.1002/(ISSN)1520-6394 11098410

[23] Araujo L Goldberg P Eyma J Madhusoodanan S Buff DD Shamin K. The effect of anxiety and depression on completion/withdrawal status in patients admitted to substance abuse detoxification program. J Subst Abuse Treat. (1996) 13:61–6. doi: 10.1016/0740-5472(95)02043-8 8699544

[24] Faravelli C. Childhood stressful events, HPA axis and anxiety disorders. World J Psychiatry. (2012) 2:13. doi: 10.5498/wjp.v2.i1.13 24175164 PMC3782172

[25] Stephens MAC Wand G. Stress and the HPA axis: role of glucocorticoids in alcohol dependence. Alcohol Res. (2012) 34:468–83. 10.35946/arcr.v34.4.11 PMC3860380 23584113

[26] Tafet GE Bernardini R. Psychoneuroendocrinological links between chronic stress and depression. Prog Neuropsychopharmacol Biol Psychiatry. (2003) 27:893–903. doi: 10.1016/S0278-5846(03)00162-3 14499305

[27] Hasler F Grimberg U Benz MA Huber T Vollenweider FX. Acute psychological and physiological effects of psilocybin in healthy humans: a double-blind, placebo-controlled dose?effect study. Psychopharmacol (Berl). (2004) 172:145–56. doi: 10.1007/s00213-003-1640-6 14615876

[28] Maillard A Cabé N Viader F Pitel AL. Neuropsychological deficits in alcohol use disorder. In: Cognition and Addiction. San Diego, California, United States: Elsevier (2020). p. 103–28. doi: 10.1016/B978-0-12-815298-0.00008-3

[29] Castaneda AE Tuulio-Henriksson A Marttunen M Suvisaari J Lönnqvist J. A review on cognitive impairments in depressive and anxiety disorders with a focus on young adults. J Affect Disord. (2008) 106:1–27. doi: 10.1016/j.jad.2007.06.006 17707915

[30] Miegel F Jelinek L Moritz S. Dysfunctional beliefs in patients with obsessive-compulsive disorder and depression as assessed with the Beliefs Questionnaire (BQ). Psychiatry Res. (2019) 272:265–74. doi: 10.1016/j.psychres.2018.12.070 30594759

[31] Korn CW Sharot T Walter H Heekeren HR Dolan RJ. Depression is related to an absence of optimistically biased belief updating about future life events. Psychol Med. (2014) 44:579–92. doi: 10.1017/S0033291713001074 PMC3880066 23672737

[32] Maurage P de Timary P Moulds ML Wong QJJ Collignon M Philippot P. Maladaptive social self-beliefs in alcohol-dependence: A specific bias towards excessive high standards. PloS One. (2013) 8:e58928. doi: 10.1371/journal.pone.0058928 23520543 PMC3592810

[33] de Vos CMH Mason NL Kuypers KPC. Psychedelics and neuroplasticity: a systematic review unraveling the biological underpinnings of psychedelics. Front Psychiatry. (2021) 12:724606. doi: 10.3389/fpsyt.2021.724606 34566723 PMC8461007

[34] Olson DE. Psychoplastogens: A promising class of plasticity-promoting neurotherapeutics. J Exp Neurosci. (2018) 12:117906951880050. doi: 10.1177/1179069518800508 PMC6149016 30262987

[35] Ly C Greb AC Cameron LP Wong JM Barragan EV Wilson PC. Psychedelics promote structural and functional neural plasticity. Cell Rep. (2018) 23:3170–82. doi: 10.1016/j.celrep.2018.05.022 PMC6082376 29898390

[36] Aleksandrova LR Phillips AG. Neuroplasticity as a convergent mechanism of ketamine and classical psychedelics. Trends Pharmacol Sci. (2021) 42:929–42. doi: 10.1016/j.tips.2021.08.003 34565579

[37] Flanagan TW Nichols CD. Psychedelics as anti-inflammatory agents. Int Rev Psychiatry. (2018) 30:363–75. doi: 10.1080/09540261.2018.1481827 30102081

[38] Attwells S Setiawan E Wilson AA Rusjan PM Mizrahi R Miler L. Inflammation in the neurocircuitry of obsessive-compulsive disorder. JAMA Psychiatry. (2017) 74:833. doi: 10.1001/jamapsychiatry.2017.1567 28636705 PMC5710556

[39] Kohler O Krogh J Mors O Eriksen Benros M. Inflammation in depression and the potential for anti-inflammatory treatment. Curr Neuropharmacol. (2016) 14:732–42. doi: 10.2174/1570159X14666151208113700 PMC5050394 27640518

[40] Zago A Pedrotti Moreira F. Alcohol use disorder and inflammatory cytokines in a population sample of young adults. J Alcohol Drug Depend. (2016) 04. doi: 10.4172/2329-6488.1000236

[41] Vollenweider FX Preller KH. Psychedelic drugs: neurobiology and potential for treatment of psychiatric disorders. Nat Rev Neurosci. (2020) 21:611–24. doi: 10.1038/s41583-020-0367-2 32929261

[42] Geyer M Vollenwider F. Serotonin research: contributions to understanding psychoses. Trends Pharmacol Sci. (2008) 29:445–53. doi: 10.1016/j.tips.2008.06.006 19086254

[43] Vollenweider F. Positron emission tomography and fluorodeoxyglucose studies of metabolic hyperfrontality and psychopathology in the psilocybin model of psychosis. Neuropsychopharmacology. (1997) 16:357–72. doi: 10.1016/S0893-133X(96)00246-1 9109107

[44] Preller KH Razi A Zeidman P Stämpfli P Friston KJ Vollenweider FX. Effective connectivity changes in LSD-induced altered states of consciousness in humans. Proc Natl Acad Sci. (2019) 116:2743–8. doi: 10.1073/pnas.1815129116 PMC6377471 30692255

[45] Vollenweider FX Csomor PA Knappe B Geyer MA Quednow BB. The effects of the preferential 5-HT2A agonist psilocybin on prepulse inhibition of startle in healthy human volunteers depend on interstimulus interval. Neuropsychopharmacology. (2007) 32:1876–87. doi: 10.1038/sj.npp.1301324 17299516

[46] Gouzoulis-Mayfrank E Heekeren K Thelen B Lindenblatt H Kovar KA Sass H. Effects of the hallucinogen psilocybin on habituation and prepulse inhibition of the startle reflex in humans. Behav Pharmacol. (1998) 9:561–6. doi: 10.1097/00008877-199811000-00011 9862081

[47] Carhart-Harris RL Friston KJ. REBUS and the anarchic brain: toward a unified model of the brain action of psychedelics. Pharmacol Rev. (2019) 71:316–44. doi: 10.1124/pr.118.017160 PMC6588209 31221820

[48] Preller KH Burt JB Ji JL Schleifer CH Adkinson BD Stämpfli P. Changes in global and thalamic brain connectivity in LSD-induced altered states of consciousness are attributable to the 5-HT2A receptor. Elife. (2018) 7. doi: 10.7554/eLife.35082 PMC6202055 30355445

[49] Dubois J VanRullen R. Visual trails: do the doors of perception open periodically? PloS Biol. (2011) 9:e1001056. doi: 10.1371/journal.pbio.1001056 21572989 PMC3091843

[50] Safron A. On the Varieties of Conscious Experiences: Altered Beliefs Under Psychedelics (ALBUS)(2020). Available online at: https://osf.io/preprints/psyarxiv/zqh4b?fbclid=IwAR02ISUGdgac3OFGn2tA7OmXovvSEw6-otYyt2qMAaqTetq1F7_QfxhCPh8 (Accessed March 13, 2024). Preprint. 10.1093/nc/niae038 PMC11823823 39949786

[51] Fortier M Millière R Nicod IJ. Psychedelics and consciousness An interview with Robin Carhart-Harris What got you interested in the topic of psychedelics?(2017). Available online at: https://www.aliusresearch.org/uploads/9/1/6/0/91600416/carhart-harris_-_psychedelics_and_consciousness.pdf?fbclid=IwAR1TXwGacT-YSTLCm-JBkpoAaj5AVJxXdEQmOtvDmlgtJF3DzoS_zfP_FMw (Accessed March 13, 2024).

[52] Carhart-Harris RL Leech R Hellyer PJ Shanahan M Feilding A Tagliazucchi E. The entropic brain: a theory of conscious states informed by neuroimaging research with psychedelic drugs. Front Hum Neurosci. (2014) 8:20. doi: 10.3389/fnhum.2014.00020 24550805 PMC3909994

[53] Carhart-Harris RL Erritzoe D Haijen E Kaelen M Watts R. Psychedelics and connectedness. Psychopharmacol (Berl). (2018) 235:547–50. doi: 10.1007/s00213-017-4701-y 28795211

[54] Doss MK Madden MB Gaddis A Nebel MB Griffiths RR Mathur BN. Models of psychedelic drug action: modulation of cortical-subcortical circuits. Brain. (2022) 145:441–56. doi: 10.1093/brain/awab406 PMC9014750 34897383

[55] Nichols D Johnson M Nichols C. Psychedelics as medicines: an emerging new paradigm. Clin Pharmacol Ther. (2017) 101:209–19. doi: 10.1002/cpt.557 28019026

[56] Krimmel SR White MG Panicker MH Barrett FS Mathur BN Seminowicz DA. Resting state functional connectivity and cognitive task-related activation of the human claustrum. Neuroimage. (2019) 196:59–67. doi: 10.1016/j.neuroimage.2019.03.075 30954711 PMC6629463

[57] Abbass A Town J Driessen E. Intensive short-term dynamic psychotherapy: A systematic review and meta-analysis of outcome research. Harv Rev Psychiatry. (2012) 20:97–108. doi: 10.3109/10673229.2012.677347 22512743

[58] Horton DM Morrison B Schmidt J. Systematized review of psychotherapeutic components of psilocybin-assisted psychotherapy. Am J Psychother. (2021) 74:140–9. doi: 10.1176/appi.psychotherapy.20200055 34293927

[59] Strickland JC Garcia-Romeu A Johnson MW. Set and setting: A randomized study of different musical genres in supporting psychedelic therapy. ACS Pharmacol Transl Sci. (2021) 4:472–8. doi: 10.1021/acsptsci.0c00187 PMC8033606 33860177

[60] Zamaria JA. A phenomenological examination of psilocybin and its positive and persisting aftereffects. NeuroQuantology. (2016) 14:285–96. doi: 10.14704/nq.2016.14.2.943

[61] Agin-Liebes G Nielson EM Zingman M Kim K Haas A Owens LT. Reports of self-compassion and affect regulation in psilocybin-assisted therapy for alcohol use disorder: An interpretive phenomenological analysis. Psychol Addictive Behav. (2024) 38:101–13. doi: 10.1037/adb0000935 PMC10696130 37276086

[62] Garcia-Romeu A. (2017). “Psychedelics as change agents in addiction and mood disorders,” in Society for the Study of Addiction 2017 Annual Conference. Northampton, United Kingdom: Society for the Study of Addiction (2017).

[63] World Health Organization. Depressive disorder (depression)(2023). Available online at: https://www.who.int/news-room/fact-sheets/detail/depression (Accessed 19.02.2024).

[64] World Health Organization. Suicide(2023). Available online at: https://www.who.int/news-room/fact-sheets/detail/suicide (Accessed 19.02.2024).

[65] Zhdanava M Pilon D Ghelerter I Chow W Joshi K Lefebvre P. The prevalence and national burden of treatment-resistant depression and major depressive disorder in the United States. J Clin Psychiatry. (2021) 82(2). doi: 10.4088/JCP.20m13699 33989464

[66] Fava M Davidson KG. Definition and epidemiology of treatment-resistant depression. Psychiatr Clinics North America. (1996) 19:179–200. doi: 10.1016/S0193-953X(05)70283-5 8827185

[67] Rush AJ Trivedi MH Wisniewski SR Nierenberg AA Stewart JW Warden D. Acute and longer-term outcomes in depressed outpatients requiring one or several treatment steps: A STAR*D report. Am J Psychiatry. (2006) 163:1905–17. doi: 10.1176/ajp.2006.163.11.1905 17074942

[68] Goodwin GM Aaronson ST Alvarez O Arden PC Baker A Bennett JC. Single-dose psilocybin for a treatment-resistant episode of major depression. New Engl J Med. (2022) 387:1637–48. doi: 10.1056/NEJMoa2206443 36322843

[69] Carhart-Harris R Giribaldi B Watts R Baker-Jones M Murphy-Beiner A Murphy R. Trial of psilocybin versus escitalopram for depression. New Engl J Med. (2021) 384:1402–11. doi: 10.1056/NEJMoa2032994 33852780

[70] Davis AK Barrett FS May DG Cosimano MP Sepeda ND Johnson MW. Effects of psilocybin-assisted therapy on major depressive disorder. JAMA Psychiatry. (2021) 78:481. doi: 10.1001/jamapsychiatry.2020.3285 33146667 PMC7643046

[71] Gukasyan N Davis AK Barrett FS Cosimano MP Sepeda ND Johnson MW. Efficacy and safety of psilocybin-assisted treatment for major depressive disorder: Prospective 12-month follow-up. J Psychopharmacol. (2022) 36:151–8. doi: 10.1177/02698811211073759 PMC8864328 35166158

[72] Carhart-Harris RL Bolstridge M Rucker J Day CMJ Erritzoe D Kaelen M. Psilocybin with psychological support for treatment-resistant depression: an open-label feasibility study. Lancet Psychiatry. (2016) 3:619–27. doi: 10.1016/S2215-0366(16)30065-7 27210031

[73] Carhart-Harris RL Bolstridge M Day CMJ Rucker J Watts R Erritzoe DE. Psilocybin with psychological support for treatment-resistant depression: six-month follow-up. Psychopharmacol (Berl). (2018) 235:399–408. doi: 10.1007/s00213-017-4771-x PMC5813086 29119217

[74] Ross S Bossis A Guss J Agin-Liebes G Malone T Cohen B. Rapid and sustained symptom reduction following psilocybin treatment for anxiety and depression in patients with life-threatening cancer: a randomized controlled trial. J Psychopharmacol. (2016) 30:1165–80. doi: 10.1177/0269881116675512 PMC5367551 27909164

[75] Griffiths RR Johnson MW Carducci MA Umbricht A Richards WA Richards BD. Psilocybin produces substantial and sustained decreases in depression and anxiety in patients with life-threatening cancer: A randomized double-blind trial. J Psychopharmacol. (2016) 30:1181–97. doi: 10.1177/0269881116675513 PMC5367557 27909165

[76] Grob CS Danforth AL Chopra GS Hagerty M McKay CR Halberstadt AL. Pilot study of psilocybin treatment for anxiety in patients with advanced-stage cancer. Arch Gen Psychiatry. (2011) 68:71. doi: 10.1001/archgenpsychiatry.2010.116 20819978

[77] von Rotz R Schindowski EM Jungwirth J Schuldt A Rieser NM Zahoranszky K. Single-dose psilocybin-assisted therapy in major depressive disorder: a placebo-controlled, double-blind, randomised clinical trial. EClinicalMedicine. (2023) 56:101809. doi: 10.1016/j.eclinm.2022.101809 36636296 PMC9830149

[78] World Health Organization. Alcohol(2022). Available online at: https://www.who.int/news-room/fact-sheets/detail/alcohol (Accessed 19.02.2024).

[79] Bogenschutz MP Forcehimes AA Pommy JA Wilcox CE Barbosa P Strassman RJ. Psilocybin-assisted treatment for alcohol dependence: A proof-of-concept study. J Psychopharmacol. (2015) 29:289–99. doi: 10.1177/0269881114565144 25586396

[80] O’Donnell KC Mennenga SE Owens LT Podrebarac SK Baron T Rotrosen J. Psilocybin for alcohol use disorder: Rationale and design considerations for a randomized controlled trial. Contemp Clin Trials. (2022) 123:106976. doi: 10.1016/j.cct.2022.106976 36332827

[81] World Health Organization. Tobacco(2023). Available online at: https://www.who.int/news-room/fact-sheets/detail/tobacco (Accessed 19.02.2024).

[82] World Health Organization. Tobacco. Cost and Expenditures(2022). Available online at: https://www.cdc.gov/tobacco/data_statistics/fact_sheets/fast_facts/cost-and-expenditures.html (Accessed 19.02.2024).

[83] Le Foll B Piper ME Fowler CD Tonstad S Bierut L Lu L. Tobacco and nicotine use. Nat Rev Dis Primers. (2022) 8:19. doi: 10.1038/s41572-022-00346-w 35332148

[84] Johnson MW Garcia-Romeu A Griffiths RR. Long-term follow-up of psilocybin-facilitated smoking cessation. Am J Drug Alcohol Abuse. (2017) 43:55–60. doi: 10.3109/00952990.2016.1170135 27441452 PMC5641975

[85] Goodman WK Grice DE Lapidus KAB Coffey BJ. Obsessive-compulsive disorder. Psychiatr Clinics North America. (2014) 37:257–67. doi: 10.1016/j.psc.2014.06.004 25150561

[86] Ruscio AM Stein DJ Chiu WT Kessler RC. The epidemiology of obsessive-compulsive disorder in the National Comorbidity Survey Replication. Mol Psychiatry. (2010) 15:53–63. doi: 10.1038/mp.2008.94 18725912 PMC2797569

[87] Moreno FA Wiegand CB Taitano EK Delgado PL. Safety, tolerability, and efficacy of psilocybin in 9 patients with obsessive-compulsive disorder. J Clin Psychiatry. (2006) 67:1735–40. doi: 10.4088/jcp.v67n1110 17196053

[88] Nutt DJ King LA Phillips LD. Drug harms in the UK: a multicriteria decision analysis. Lancet. (2010) 376:1558–65. doi: 10.1016/S0140-6736(10)61462-6 21036393

[89] Raval NR Johansen A Donovan LL Ros NF Ozenne B Hansen HD. A single dose of psilocybin increases synaptic density and decreases 5-HT2A receptor density in the pig brain. Int J Mol Sci. (2021) 22:835. doi: 10.3390/ijms22020835 33467676 PMC7830000

[90] Krebs TS Johansen PØ. Psychedelics and mental health: A population study. PloS One. (2013) 8:e63972. doi: 10.1371/journal.pone.0063972 23976938 PMC3747247

[91] Basedow LA Kuitunen-Paul S. Motives for the use of serotonergic psychedelics: A systematic review. Drug Alcohol Rev. (2022) 41:1391–403. doi: 10.1111/dar.13480 35668698

[92] Newson M Khurana R Cazorla F van Mulukom V. ‘I get high with a little help from my friends’ - how raves can invoke identity fusion and lasting co-operation via transformative experiences. Front Psychol. (2021) 12. doi: 10.3389/fpsyg.2021.719596 PMC8504457 34646208

[93] Gordon AR Carrithers BM Pagni B Kettner H Marrocu A Nayak S. The Effect of Psychedelics on Individuals with a Personality Disorder: Results from two Prospective Cohort Studies(2024). Available online at: https://www.researchsquare.com/article/rs-4203641/v1 (Accessed 07, 2024). Preprint.

[94] Wolf G Singh S Blakolmer K Lerer L Lifschytz T Heresco-Levy U. Could psychedelic drugs have a role in the treatment of schizophrenia? Rationale and strategy for safe implementation. Mol Psychiatry. (2023) 28:44–58. doi: 10.1038/s41380-022-01832-z 36280752

[95] Sandberg E. Psilocybin Psychotherapy with Acceptance and Commitment Therapy: A Potential Treatment for Social Anxiety Disorder (2022). Belmont University Research Symposium (BURS. Available online at: https://repository.belmont.edu/burs/91 (Accessed May 7, 2024).

[96] Biscoe N Bonson A Slavin M Busuttil W MacManus D Cox A. Psilocybin-assisted psychotherapy for the treatment of PTSD in UK armed forces veterans: A feasibility study protocol. Eur J Trauma Dissociation. (2023) 7:100359. doi: 10.1016/j.ejtd.2023.100359

[97] Koning E Brietzke E. Psilocybin-assisted psychotherapy as a potential treatment for eating disorders: a narrative review of preliminary evidence. Trends Psychiatry Psychother. (2023). doi: 10.47626/2237-6089-2022-0597 PMC11790131 37126863

[98] Zeifman RJ Kettner H Pagni BA Mallard A Roberts DE Erritzoe D. Co-use of MDMA with psilocybin/LSD may buffer against challenging experiences and enhance positive experiences. Sci Rep. (2023) 13:13645. doi: 10.1038/s41598-023-40856-5 37608057 PMC10444769

[99] Chen T Cheng L Ma J Yuan J Pi C Xiong L. Molecular mechanisms of rapid-acting antidepressants: New perspectives for developing antidepressants. Pharmacol Res. (2023) 194:106837. doi: 10.1016/j.phrs.2023.106837 37379962

[100] Włodarczyk A Słupski J Szarmach J Cubała WJ. Single arketamine in treatment resistant depression: Presentation of 3 cases with regard to sick-leave duration. Asian J Psychiatr. (2024) 96:104016. doi: 10.1016/j.ajp.2024.104016 38554563

[101] Sekula AD Downey L Puspanathan P. Virtual reality as a moderator of psychedelic-assisted psychotherapy. Front Psychol. (2022) 13. doi: 10.3389/fpsyg.2022.813746 PMC8931418 35310225

[102] Smigielski L Scheidegger M Kometer M Vollenweider FX. Psilocybin-assisted mindfulness training modulates self-consciousness and brain default mode network connectivity with lasting effects. Neuroimage. (2019) 196:207–15. doi: 10.1016/j.neuroimage.2019.04.009 30965131

[103] Wolff M Evens R Mertens LJ Koslowski M Betzler F Gründer G. Learning to let go: A cognitive-behavioral model of how psychedelic therapy promotes acceptance. Front Psychiatry. (2020) 11:5. doi: 10.3389/fpsyt.2020.00005 32153433 PMC7046795

[104] Watts R Luoma JB. The use of the psychological flexibility model to support psychedelic assisted therapy. J Contextual Behav Sci. (2020) 15:92–102. doi: 10.1016/j.jcbs.2019.12.004

[105] Brennan W Belser AB. Models of psychedelic-assisted psychotherapy: A contemporary assessment and an introduction to EMBARK, a transdiagnostic, trans-drug model. Front Psychol. (2022) 13:866018. doi: 10.3389/fpsyg.2022.866018 35719571 PMC9201428

[106] Sloshower J Guss J Krause R Wallace RM Williams MT Reed S. Psilocybin-assisted therapy of major depressive disorder using Acceptance and Commitment Therapy as a therapeutic frame. J Contextual Behav Sci. (2020) 15:12–9. doi: 10.1016/j.jcbs.2019.11.002

[107] Chambers R Stoliker D Simonsson O. Psychedelic-assisted psychotherapy and mindfulness-based cognitive therapy: potential synergies. Mindfulness (N Y). (2023) 14:2111–23. doi: 10.1007/s12671-023-02206-4

[108] MacCallum CA Lo LA Pistawka CA Deol JK. Therapeutic use of psilocybin: Practical considerations for dosing and administration. Front Psychiatry. (2022) 13:1040217. doi: 10.3389/fpsyt.2022.1040217 36532184 PMC9751063

[109] Perez N Langlest F Mallet L De Pieri M Sentissi O Thorens G. Psilocybin-assisted therapy for depression: A systematic review and dose-response meta-analysis of human studies. Eur Neuropsychopharmacol. (2023) 76:61–76. doi: 10.1016/j.euroneuro.2023.07.011 37557019

[110] Yerubandi A Thomas JE Bhuiya NMMA Harrington C Villa Zapata L Caballero J. Acute adverse effects of therapeutic doses of psilocybin. JAMA Netw Open. (2024) 7:e245960. doi: 10.1001/jamanetworkopen.2024.5960 38598236 PMC11007582

[111] Gard DE Pleet MM Bradley ER Penn AD Gallenstein ML Riley LS. Evaluating the risk of psilocybin for the treatment of bipolar depression: A review of the research literature and published case studies. J Affect Disord Rep. (2021) 6:100240. doi: 10.1016/j.jadr.2021.100240

